# Olympic Glory Vs. Athlete Safety: Ethical Lessons From the Seine Water Quality Controversy

**DOI:** 10.3389/phrs.2024.1608075

**Published:** 2024-11-19

**Authors:** Giulia Sesa, Pascal Borry, Sigmund Loland, Silvia Camporesi

**Affiliations:** ^1^ Centre for Biomedical Ethics and Law, Department of Public Health and Primary Care, Faculty of Medicine, KU Leuven, Leuven, Belgium; ^2^ Department of Sport and Social Sciences, Norwegian School of Sport Sciences, Oslo, Norway; ^3^ Department of Public Health and Primary Care, Centre for Biomedical Ethics and Law, Faculty of Movement and Rehabilitation Sciences, KU Leuven, Leuven, Belgium

**Keywords:** public health, ethics, olympics, athletes’ safety, sporting events

## Paris 2024 and the Case of the River Seine

Sports are often celebrated for promoting health and wellbeing. However, the controversy over the Seine’s water quality during the Paris 2024 Olympics and Paralympics highlights the stark contrast between this idealized vision and the reality of health risks athletes face. After Belgian triathlete Claire Michel was hospitalized due to an E. Coli infection, Belgium withdrew its triathlon team [1]. Athletes from other countries also experienced gastrointestinal symptoms, possibly linked to bacteria in the Seine. The murky water also posed visibility challenges, raising concerns over wounds caused by brambles. Some athletes attempted to minimize their risk of infection by taking probiotics, while others took drastic measures that endangered their health, such as intentionally exposing themselves to *E. Coli* [2]. These incidents raise ethical tensions around ensuring athlete safety, safeguarding public health, and the international pressure of hosting high-profile sporting events.

## Failures in Upholding Athletes’ Safety

The 2024 Paris Olympics and Paralympics swimming events held in the River Seine presented significant challenges in ensuring athlete safety and maintaining the integrity of the competition environment.

A key issue was the lack of transparency in the decision-making process concerning water quality standards and the decision to allow competitions. Delayed reporting of water quality data and limited evidence to support safety claims, were significant problems during the Games [3, 4]. Furthermore, the criteria used to determine whether athletes could safely compete were not adequately justified. For example, the World Triathlon Water Quality Statement advises triathlons to occur in waters where *E. coli* levels remain below 500 CFU/100 mL and Enterococci under 200 CFU/100 mL [5]. However, for the Paris 2024 Olympics, these limits were raised significantly, with *E. coli* levels set at 1,000 CFU/100 mL and Enterococci at 400 CFU/100 mL [4]. Such deviations from established standards warrant clear, transparent justifications, but these were either not provided or inadequately communicated to the public. The absence of a centralized platform where water quality reports were publicly accessible only exacerbated these issues.

Additionally, the final call on allowing swimming in the Seine during the triathlon, paratriathlon, and open water Olympic events, was made by the relevant sport federation, viz. World Triathlon or World Aquatics, in coordination with the Paris 2024 Organizing Committee. While procedures followed EU and WHO standards [5], sport bodies’ decisions risk prioritizing event schedules, reputations, and legacies over health concerns. Moreover, if federations primarily conduct risk assessments without transparent criteria it further complicates the credibility of these decisions.

Another significant issue was the inadequacy of strategies to minimize health risks during the events. While strategies such as water testing were a step in the right direction, individual health precautions were insufficient. Athletes lacked clear guidance on reducing health risks and took measures like reducing handwashing and attempting to avoid swallowing water during swimming to prevent infection [2]. Furthermore, misconceptions about health risks can skew our understanding of acceptable risks. In the sports context potential infection spread is often overlooked [6]. Moreover, the portrayal of athletes as enduring heroes [7], can lead to compromising their safety. Most importantly, the challenge of establishing a direct link between an infection and exposure to contaminated water further complicates the understanding of the risks athletes face [5].

Compounding these issues was the lack of consideration or clear communication about alternative venues or contingency plans, leaving it unclear whether alternative options were genuinely explored. The decision to hold competitions in the Seine, despite its questionable water quality, appeared non-negotiable, with no alternatives considered, reflecting a mindset of “*either the Seine or death*” as stated by Italian coach Stefano Rubaudo [8]. This choice suggests a prioritization of image and global prestige, underscored by the Seine’s symbolic importance as part of Paris’s Olympic legacy, over athlete safety [4].

Ultimately, are challenges that extend beyond the capabilities of sport federations and reflect broader difficulties in assessing water quality. While *E. coli* and *Enterococcus* are frequently used to assess water quality, there are concerns that this focus may not fully capture the extent of sewage contamination [9]. Water quality fluctuations due to weather conditions add further difficulties. Fluidion, a water intelligence company, has criticized current water quality assessment methods for underestimating actual contamination and provided data on the Seine’s water quality using novel techniques [10]. Novel techniques lack international validation, raising the question of whether emerging evidence should be considered if available.

## Balancing Athlete Safety, Public Health, and the Drive to Host High-Profile Sporting Events: Key Ethical Considerations

To strike an ethical balance between the safety of athletes, public health concerns, and the aspiration to conduct high-profile sporting events, it is imperative to incorporate fundamental principles of public health ethics into the decision-making process.

First and foremost, it is essential to provide clear and transparent information about the rationale behind decisions and the processes involved in determining the safety of competition sites. Timely disclosure of information in an understandable language, combined with honest and truthful communication allows stakeholders, including athletes, coaches, and the public, to understand the potential risks associated with competition. While fully informed and autonomous decisions may be challenging in the sports context [11], transparency helps foster and sustain trust within the sporting community.

Independent oversight and adherence to scientific standards are essential when making decisions related to athletes’ safety. While multiple stakeholders should be involved in the decision-making process, the final determination on whether to proceed with events amid health risks should be made by independent public health authorities rather than sports federations. This ensures that athlete health is prioritized. Additionally, since the final decision affects athletes directly, it is important to include athletes’ committees, comprised of current or former athletes, in the decision-making process, ensuring procedural justice.

Accurate risk assessments, along with practical and effective risk minimization and mitigation strategies are crucial in guaranteeing the safety of competition sites and in finding a balance between the desire to host the event and costs of allowing participation. When the safety of a venue remains uncertain, or risk minimization or mitigation strategies remain impractical, contingency plans should be thoroughly thought of and publicly disclosed.

Ultimately, cities and governments should routinely prioritize public health and environmental issues to ensure a fairer and more transparent approach to hosting major sporting events. The Rio 2016 Olympics underscored how critical issues like water contamination—affecting millions daily—were only addressed due to the event, despite years of protests [9]. If improvements are made they must be long-lasting or they will merely waste resources that could have been invested in areas with more lasting benefits.

## Reflections and Recommendations for LA2028

The safety of competition environments has been a recurring concern. Issues with water quality were prominent at the Sydney 2000, Beijing 2008, London 2012, and Rio 2016 Olympics. Similar challenges are anticipated for the upcoming Los Angeles Olympics. While exposure to such risks may be considered an inherent part of the sport it is essential to reflect on whether effective measures and procedures exist to effectively minimize them.

As we look ahead to the LA 2028 Summer Olympics, we hope that the considerations presented in this commentary can assist in better navigating the complex challenges of safeguarding athletes’ health and upholding the safety of competition environments also beyond urban waterways.
